# Accuracy Enhancement of Anomaly Localization with Participatory Sensing Vehicles

**DOI:** 10.3390/s20020409

**Published:** 2020-01-11

**Authors:** Raj Bridgelall, Denver Tolliver

**Affiliations:** 1 Department of Transportation, Logistics, & Finance, North Dakota State University, Department 2880 P.O. Box 6050, Fargo, ND 58101, USA; 2 Upper Great Plains Transportation Institute, North Dakota State University, Department 2880 P.O. Box 6050, Fargo, ND 58101, USA; denver.tolliverl@ndsu.edu

**Keywords:** crowdsourced sensing, GPS tagging latency, GPS errors, GPS resolution, low-cost GPS, pothole detection, roadway anomalies, railway anomalies, standard positioning service

## Abstract

Transportation agencies cannot afford to scale existing methods of roadway and railway condition monitoring to more frequently detect, localize, and fix anomalies throughout networks. Consequently, anomalies such as potholes and cracks develop between maintenance cycles and cause severe vehicle damage and safety issues. The need for a lower-cost and more-scalable solution spurred the idea of using sensors on board vehicles for a continuous and network-wide monitoring approach. However, the timing of the full adoption of connected vehicles is uncertain. Therefore, researchers used smartphones to evaluate a variety of methods to implement the application using regular vehicles. However, the poor accuracy of standard positioning services with low-cost geospatial positioning system (GPS) receivers presents a significant challenge. The experiments conducted in this research found that the error spread can exceed 32 m, and the mean localization error can exceed 27 m at highway speeds. Such large errors can make the application impractical for widespread use. This work used statistical techniques to inform a model that can provide more accurate localization. The proposed method can achieve sub-meter accuracy from participatory vehicle sensors by knowing only the mean GPS update rate, the mean traversal speed, and the mean latency of tagging accelerometer samples with GPS coordinates.

## 1. Introduction

Roadway and railway networks are important arteries that support the economic well-being of a nation. These networks require regular maintenance because of continuous deterioration from usage and weather cycles. The development of roadway anomalies such as potholes, frost heaves, swelling, rutting, bumps, sags, and cracks can slow traffic, cause ride discomfort, damage vehicles, and create safety issues [1]. However, transportation agencies cannot afford to monitor the condition of roadways and railways more frequently than needed because of the high cost and slow speed of current methods. Current standard practices require specially equipped profiler vehicles, skilled personnel to operate them, and time-intensive post-processing to classify the ride quality [2]. The profiler vehicles use geospatial positioning system (GPS) receivers that update 20 times faster than the low-cost GPS receivers in smartphones, and they use real-time inertial measurement units (IMUs) to improve position estimates [2]. Even so, the profiler vehicles still cannot accurately estimate the roughness of low-speed and urban roads [3]. Hence, these limitations spurred research to develop alternative means of achieving more automated, frequent, and network-wide monitoring. This naturally led to the idea of using regular vehicles as participatory sensors [4,5]. However, there is a gap in research with regard to characterizing the localization accuracy of a participatory sensing method.

### 1.1. Literature Review

Previous research established that the minimum onboard devices needed to implement anomaly detection and localization include a GPS receiver, an accelerometer, a speed sensor, a timer, a wireless internet transceiver, a microprocessor with digital memory, and a reliable power source [5]. Connected vehicles already integrate and provide access to such devices, but their full adoption is uncertain. There were many studies exploring methods of roughness classification using smartphones on board regular vehicles because they can provide the same capability as connected vehicles sensors [6,7,8]. When used on connected locomotives, the method can prevent track capacity loss from closures during condition monitoring with specially equipped railway vehicles [9,10,11]. The device orientation may be fixed when using smartphone sensors to emulate this future probe vehicle application. However, methods that intend to use smartphones that are not in a fixed position during the sensing application may apply available methods to estimate their pose if needed [12].

One disadvantage of the participatory sensing approach is that the integrated GPS receiver of vehicles and smartphones provides a standard positioning service (SPS), where the accuracy and resolution are much lower than those of the specialized monitoring vehicles [13]. Given this limitation of low-cost GPS, there is a surprising gap in the literature regarding studies to characterize localization errors in a participatory sensing application. Most studies focused on evaluating related problems of characterizing the accuracy of roughness intensity measurements [14,15], approximating the international roughness index (IRI) [16,17], or classifying segment roughness using machine learning techniques [18,19]. A related finding from evaluations of commercial smartphone applications (apps) is that they disagree in IRI estimates and provide inconsistent results [20].

The global average horizontal error of the SPS was recently measured as approximately 9 m within a 95% confidence interval [13]. However, those measurements excluded errors due to atmospheric conditions, receiver errors, multipath reflections, terrain masking, and foliage. Testing in urban environments demonstrated that multipath reflections could double the ground-truth distances [21]. A literature search as of the time of this writing did not find a study that characterized the anomaly localization error from using low-cost GPS receivers on board regular vehicles.

### 1.2. Goals and Objectives

**Figure 1:**
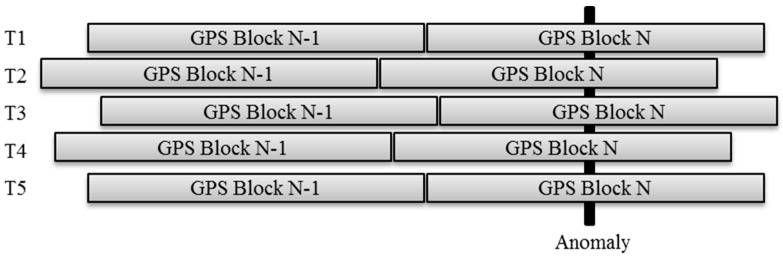
The position of geospatial positioning system (GPS) blocks from multiple traversals.

The goal of this research was to characterize the position accuracy of localizing roadway and railway anomalies by using widely available low-cost sensors. Consequently, the authors developed an iOS^®^ app and an Android app called PAVVET and RIVET, respectively, which can access all the required sensors on a smartphone [22]. These apps include smart power management, remote sensing, security features, and other modes that were needed for this and other projects. The standard GPS receiver of smartphones updates at a rate of 1 Hz, whereas the accelerometer samples at a rate of more than 90 Hz, depending on the device model. This difference in update rates causes the apps to tag a block of accelerometer samples with the same GPS coordinates. For example, if the accelerometer updates 90 times per second but the GPS receiver updates only once per second, the system will tag all 90 accelerometer samples with the GPS coordinates from its last update. Figure 1 illustrates how the resolution gap creates blocks of accelerometer samples with the same geospatial position. Hence, in addition to errors in the geospatial positions reported, the low update rate of low-cost GPS receivers causes a position resolution gap. Table 1 shows an example of the data collected for a GPS block that contains the peak accelerometer signal Gz highlighted as a g-force value of −1.216 at time instant of 46.768 s. This is called a peak inertial event (PIE).

GPS blocks update asynchronously along the traversal path. Therefore, the geospatial position updates among traversals T1, T2, …, TN will occur at different geospatial positions. Traversing an isolated anomaly will produce a PIE within a GPS block. As shown in Figure 1, the position of the PIE within a GPS block mirrors the asynchronous GPS updates.

**Figure 2:**
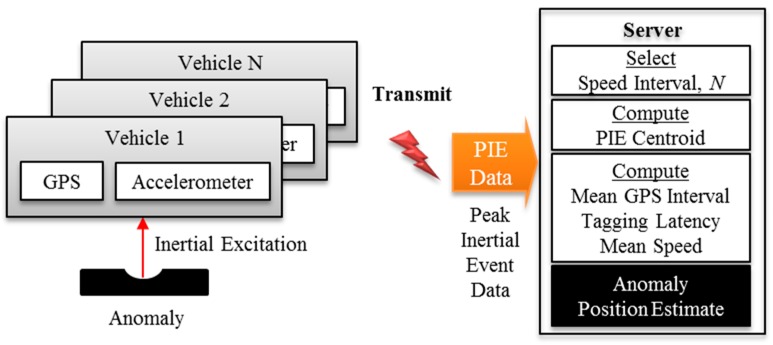
Graphical overview of the approach.

The main contribution of this work is a method to estimate the *position* of an anomaly from a hotspot of the low-accuracy and low-resolution GPS coordinates of the associated PIE detected. Previous work addressed the complementary problem of anomaly *detection* by using the participatory sensing approach to enhance signal quality [23]. That is, anomaly detection must occur before estimating its position. This paper addresses the latter. The experiments conducted showed that it is possible to achieve sub-meter localization accuracy from participatory sensors by knowing only the mean GPS update rate, the mean traversal speed, and the mean latency of tagging accelerometer samples with GPS coordinates. Figure 2 is a graphical abstract of the approach. An additional pavement management application (not shown) is needed for a complete solution to inform regular road maintenance. For example, the anomaly position estimate software can feed its output to existing pavement management systems that require the positions of anomalies as inputs.

The remainder of this paper is organized into four additional sections. Section 2 develops the error model of localization and describes the experiment design to evaluate the statistical nature of the error components. Section 3 provides the results from the experiments and determines the goodness-of-fit of hypothesized distributions of the error components. Based on observations of those error distributions, Section 4 develops a method to estimate the position of an anomaly from many GPS tags of the accelerometer sample associated with the anomaly. Section 5 provides some concluding remarks about the findings and briefly describes future work to address a shortcoming of the approach.

## 2. Materials and Methods

This section of the paper develops the error model and describes the experiments designed to characterize the statistical nature of the error components.

### 2.1. Error Model

The geodesic distance between the coordinates of the GPS block containing the PIE and the ground truth (*D*_GT_) has three components: (1) a distance error *D_RES_* due to the GPS position resolution gap, (2) the sensor distance offset *D*_AX_ from the axle of the vehicle that generates the PIE, and (3) any residual error *d*_ε_, which is currently unknown. Hence, the distance error model is

(1) $$D_{\mathrm{GT}}=D_{\mathrm{RES}}+D_{\mathrm{AX}}+d_{\varepsilon }$$

where only one parameter *D*_AX_ is deterministic. It is possible to determine the distribution of the GPS distance error *D*_GT_ and the GPS position resolution gap *D*_RES_ with a specially designed experiment. Consequently, the residual error *d*_ε_ is a function of those two distributions, such that

(2) $$d_{\varepsilon }=D_{\mathrm{GT}}-D_{\mathrm{RES}}-D_{\mathrm{AX}}$$

The sign convention is that a positive distance value will be ahead of the anomaly position (ground truth) in the direction of travel. Therefore, an accelerometer placed at a position behind the front axle would be at a negative distance offset when tracking the PIE generated by the front axle, and a positive distance offset when tracking the PIE generated by the rear axle.

The ground truth was selected as a spot on the anomaly that was directly below the position where the smartphones crossed. The geospatial coordinates of the spot were determined with a more accurate device used for land surveying and verified with commercial-grade geographic information system (GIS) software. The GPS distance error *D*_GT_ is the geodesic distance between the ground truth and the GPS tag of the PIE. The geodesic distance is the shortest distance between two points on the surface of an ellipsoid model of Earth. The method of Vincenty (1975) or Karney (2013) can compute the geodesic distance, but the latter guarantees convergence for nearly antipodal points [24]. The authors modified the Karney (2013) method to produce negative distances in the direction of travel for points reported behind the ground truth, and positive distances otherwise.

The GPS resolution gap distance for a traversal is

(3) $$D_{RES}=\left(t_{p}-t_{b}\right)S_{p}=T_{RES}S_{p}$$

where *t*_p_ is the time instant associated with the PIE, *t*_b_ is the time instant of the beginning of the GPS block containing the PIE, T_RES_ is the associated GPS resolution gap time, and *S*_p_ is the average speed of the vehicle during the GPS block containing the PIE.

### 2.2. Experiment Design

The experiments covered a diverse combination of factors that could potentially affect GPS accuracy with the SPS. Factors included the environment, sensor model, vehicle type, sensor placement, vehicle speed, and the type of anomaly. Table 2 summarizes the characteristics of the equipment used to collect data from 12 sets of traversals in different vehicles and environments. The experiments used six different phone models, five different traversal speeds, four different environments, three different vehicle types, and three different sensor mounts. Experiments in two different environments used the same vehicle, sensor mount, smartphone models, vehicle speed, and type of isolated roughness generator. The vehicle maintained a constant speed for each set of traversals to minimize any variability due to speed. The Park Road experiment used the same equipment but maintained a different speed for three sets of traversals. For that experiment, the smartphone was mounted behind the position of the front axle that generated the PIE. For the MnRoad experiment, the smartphone was mounted behind the position of the rear axle that generated the PIE. For the Unpaved/Paved Road experiments, the smartphone was mounted ahead of the position of the rear axle that generated the PIE. In all cases, the sensor was secured to a flat surface of the vehicle’s body for maximum vibration coupling. Tape held the devices securely to the surface to prevent spurious vibrations from preventing consistent detection of the desired PIE signal.

**Figure 3:**
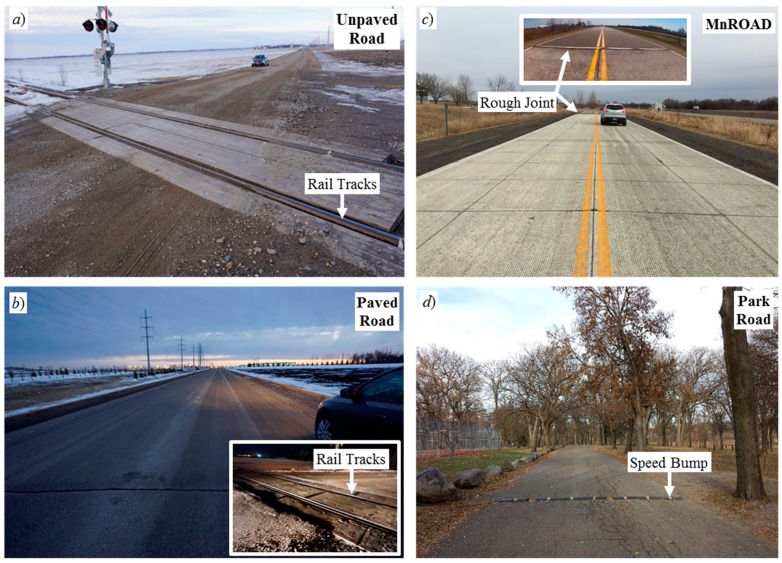
Environments of the (**a**) Unpaved Road, (**b**) Paved Road, (**c**) MnROAD, and (**d**) Park Road.

The isolated roughness generator produced a PIE that was easy to detect accurately within the accelerometer signal sample stream. Figure 3 shows the nature of each environment and their isolated roughness generators. Figure 3a,b shows the rail-grade crossings that generated the PIEs for the Unpaved Road and Paved Road traversals, respectively. The inset of Figure 3c displays the rough joint between the asphalt and concrete pavements that generated the PIEs of the MnROAD traversals. Figure 3d shows the speed bump that produced the PIEs of the Park Road traversals.

**Figure 4:**
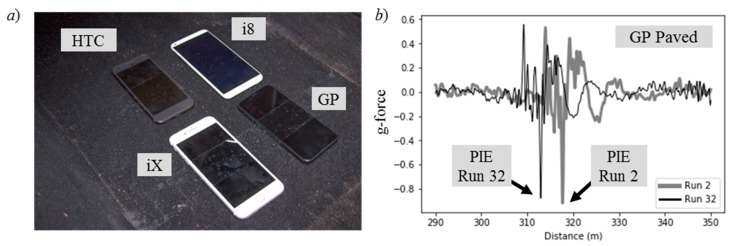
(**a**) Smartphones secured to the sedan floor, and (**b**) two accelerometer signals with peak inertial event (PIEs).

Figure 4a shows the smartphones mounted to the floor of the sedan used for the Unpaved and Paved Road traversals. The smartphones were positioned just behind the front passenger seat, midway between the two axles of the sedan. Both the Unpaved and the Paved Road experiments collected data with two smartphones running the Android^®^ operating system (HTC and Google Pixel (GP)) and two running the iOS^®^ operating system (i8 and iX) from Apple, Inc. Unfortunately, the HTC did not record GPS coordinates for any of the traversals; thus, the data were consequently abandoned.

Figure 4b plots a portion of the accelerometer signal from the GP smartphone that contained the PIEs from runs two and 32 of the Paved Road traversals. The distance label on the horizontal axis is the interpolated distance from a common geospatial reference line C0 that bisects the traversal path. The interpolated distance is

(4) $$d_{n}=d_{n-1}+v_{n}\times \Delta \tau_{n}$$

where *n* is the sample number, *v_n_* is the instantaneous speed logged for that sample instant, and Δ*τ_n_* is the sample period at that sample instant. The initial position *d*_0_ = 0 is the distance interpolated position that was closest to C0. This position was determined by firstly identifying the GPS block located prior to C0 and then interpolating the path distance from the first sample of that GPS block until reaching the signal sample that was closest to C0. The observed misalignment of the PIEs among traversals was due to the position error of the coordinates reported for the GPS block closest to C0.

## 3. Results

**Figure 5:**
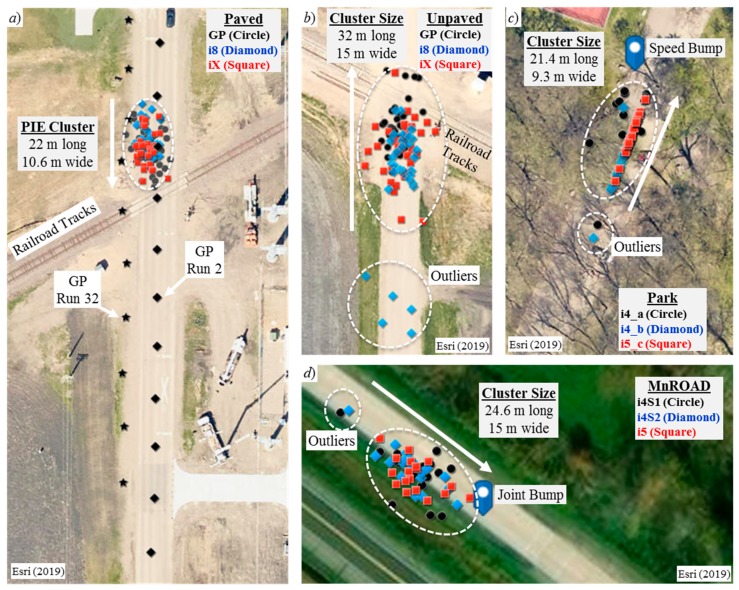
PIE clusters: (**a**) Paved Road, (**b**) Unpaved Road, (**c**) Park Road, and (**d**) MnROAD.

Figure 5 shows the relative geospatial positions of the PIEs from all traversals of each roughness generator. Each circle, diamond, or square shown in Figure 5 represents a PIE from the traversals of one device or experiment as indicated. The insets label the dimensions of the elliptically shaped PIE clusters. The outliers are indicated within the circular boundaries. Table 3 summarizes the values of the various parameters derived from the experiments.

The roughness generators labeled as shown were the railroad tracks, the speed bump, and the joint bump. The arrow next to each PIE cluster indicates the direction of travel. It is evident that, regardless of the travel direction, the centers of each PIE cluster were consistently located prior to the roughness generators. Figure 5a complements Figure 4b by showing how the geospatial coordinate updates for the GPS blocks to the two traversals are spatially asynchronous. The figure shows the positions of each GPS block along the traversal path of the GP device. The GPS coordinates for run 32 exited the traversal path even though the vehicle remained on the path.

Table 3 lists the number of traversals conducted for each experiment as *N*. Statisticians often consider more than 30 trials to be statistically significant [25]. Even with 18 MnROAD traversals, the GPS distance distributions converged to a typical bell-shaped curve, which facilitated hypothesis testing with a normal distribution. The apps configured the smartphones to sample at their highest rate. The iOS^®^ models sampled between 91 and 134 Hz, whereas the GP sampled at approximately 385 Hz. The mean sample rate of the GP differed by approximately 1 Hz among the Paved and Unpaved Road experiments. This is due to the statistical nature of the sampling, as discussed in previous work [23]. The authors plan to publish the collected data in a future journal article [26].

### 3.1. Error Distribution

**Figure 6:**
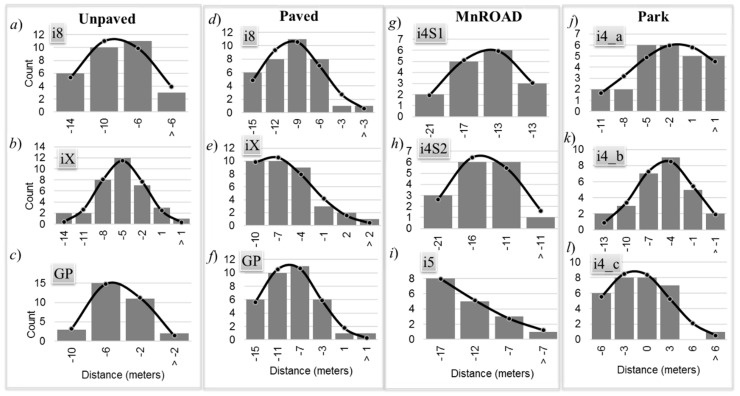
Distribution of GPS distance error.

Figure 6 shows the histogram of the GPS distance error *D_GT_* for each experiment as bar charts. The line graph is the best-fit normal distribution to the histogram.

The best fit was determined by solving the following optimization problem:

(5) $$\underset{X_{i}}{\mathrm{minimize}}\;e=\overset{B}{\underset{i=1}{\sum }}{\left(H_{i}-D_{i}\right)}^{2},\;\mathrm{subject}\;\mathrm{to}\;\alpha >0,\;\sigma >0,\;\mathrm{and}\;N\geq B\geq 4,\;\mathrm{where}\;D_{i}=\frac{\alpha }{\sqrt{2\pi \sigma^{2}}}e^{-\frac{{\left(X_{i}-\mu \right)}^{2}}{2\sigma^{2}}},\;i=\;1,\;2,\;\ldots ,\;B$$

The counts for values that fall within interval *X_i_* of histogram bin *i* are *H_i_*. The values of the distribution evaluated at interval *X_i_* are *D_i_* where the parameters that minimize the sum-of-squares (SOS) error e are the amplitude *α*, mean *µ*, and variance *σ*^2^. A Pearson’s chi-squared test quantified the goodness-of-fit of the solution by computing the chi-squared statistic.

(6) $$\chi_{k}^{2}=\overset{B}{\underset{i=1}{\sum }}\frac{{\left(H_{i}-D_{i}\right)}^{2}}{D_{i}}$$

The parameter *k* represents the degrees of freedom (*df*) in statistics. The value of *k* associated with the chi-squared statistic is the number of histogram bins minus the three parameters *α*, *µ*, and *σ* needed to obtain the best-fit distribution. Hence, the *B* histogram bins must be at least four so that the *df* can be at least unity. The number of bins also has an upper bound because the number of bins cannot exceed the number of samples *N*.

The probability *p* that the chi-squared statistic computed is at least as large as the expected value is the area under the chi-squared distribution curve, where

(7) $$p=\int_{\chi_{k}^{2}}^{\infty }\frac{1}{2^{k/2}\Gamma \left(k/2\right)}x^{k/2-1}e^{-x/2}dx$$

and *Γ* (*k*/2) is the gamma function in mathematics. The computed probability is known as the *p*-value of the hypothesis test [25]. The most common practice is to reject the hypothesis that the distribution follows the tested distribution when the *p*-value is less than 0.05, and to fail to reject the hypothesis otherwise. Intuitively, a large difference between the observed and best-fit distributions results in a large chi-squared statistic that is associated with a low likelihood.

**Figure 7:**
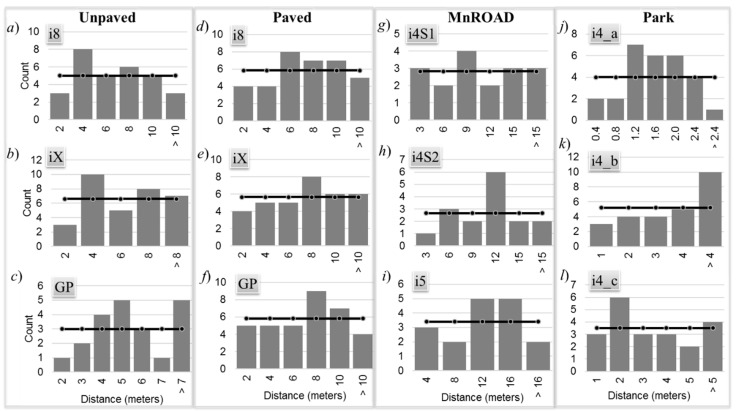
Distribution of GPS resolution gap distance.

Table 3 lists the *p*-values from the chi-squared goodness-of-fit tests of the *D*_GT_ distribution for a normal distribution. The null hypothesis is that the distributions follow a normal distribution. None of the tests could reject the hypothesis that the GPS distance error is normally distributed because the *p*-values for all experiments were greater than the standard significance of 0.05. This suggests that the alternative hypothesis could be accepted. It is difficult to observe visually that some of the distributions follow a normal distribution because of their large negative bias and large skew. The average GPS resolution gap time *T*_RES_ was 0.55 s with a standard deviation of 0.05 s. The GPS resolution gap distance was computed from Equation (3), and Figure 7 shows the distribution of those distances for each experiment.

Table 3 lists the *p*-values from the chi-squared goodness-of-fit tests of the *D*_RES_ distribution for a uniform distribution. The *df* for this test was the number of histogram bins minus one parameter α needed obtain the best-fit uniform distribution. The hypothesis was that the distributions follow a uniform distribution. Since the *p*-values for all experiments were greater than the standard significance of 0.05, none of the tests could reject the hypothesis that the GPS resolution gap distance was uniformly distributed with a positive bias.

**Figure 8:**
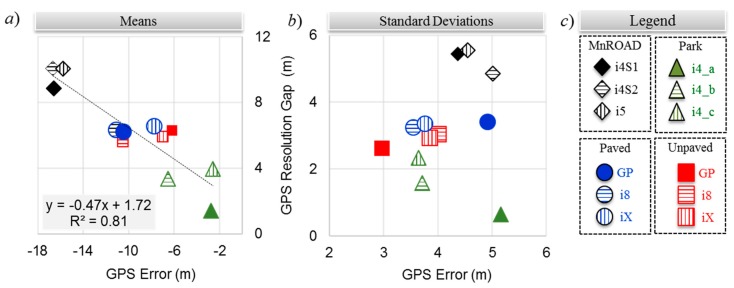
GPS ground truth offset and resolution gaps: (**a**) mean distances, (**b**) standard deviations, and (**c**) chart legend.

Figure 8a,b compares the means and standard deviations of the GPS distance error and the GPS resolution gap distances, respectively. The legend of Figure 8c helps with visualizing the error patterns among the smartphones in the four experiment environments. The mean errors tended to cluster with speed. From Table 3, the mean speeds were approximately 19, 11, and 5 m∙s^−1^ for the MnROAD, Paved/Unpaved, and Park Road experiments, respectively. Although three smartphones were in the same spot for the MnROAD, Paved, and Unpaved Road experiments, the difference in their mean errors ranged from less than 1 m to more than 4 m. Except for the highest and lowest speed traversals, the mean GPS resolution gap distance was consistent among smartphones.

Unlike the means of the distance errors, the associated standard deviations did not tend to cluster with speed. The standard deviations ranged from less than 3 m to more than 5 m. The variance was largest for the lowest-speed traversals of the Park Road experiment and the high-speed traversals of the MnROAD experiments. However, the variance for the two higher speeds of the Park Road experiment was consistent with that of the Paved Road and Unpaved Road experiments. Within each testing environment, the variance of the GPS distance error was similar among the iOS^®^ device models, but the differences were more extreme for the Android device. Except for the lowest-speed traversals of the Park Road experiment, the variance of the GPS resolution gap error was consistent among smartphones for the experiments of each environment.

### 3.2. Residual Error

**Figure 9:**
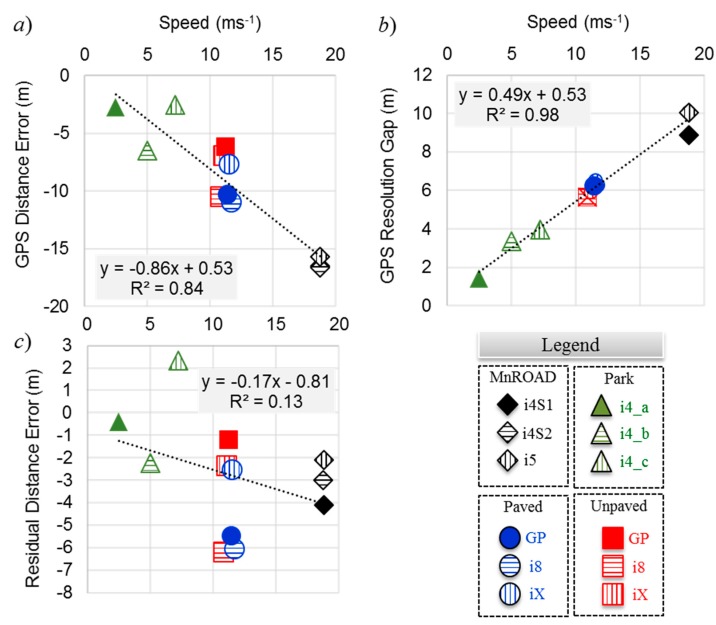
Correlation with speed for (**a**) GPS distance error, (**b**) GPS resolution gap, and (**c**) residual distance error.

As observed in Figure 9a, the GPS distance error, which is the geodesic distance error from the ground truth, varied directly with the mean traversal speed. Figure 9b shows that the GPS resolution gap distance was even more highly correlated with speed because the coefficient of determination *R*^2^ for the regression equation shown in the inset was close to unity. The correlation with speed is intuitive because the length of a GPS block must be directly proportional to the vehicle’s speed since the GPS blocks are all approximately 1 s long. The implication from the regression model is that distance errors in this application can approach 30 m at highway speeds, making it impractical to locate an anomaly within sight distance.

The mean residual distance error was determined from Equation (2) by using the mean GPS resolution gap distance. Figure 9c shows the correlation of the residual distance error with speed. Unlike the mean GPS resolution gap distance, the mean residual distance error was not correlated with the mean traversal speed—the *R*^2^ value was only 0.13. This is evidence that the residual distance error must be due to a source that is not related to the movement of the vehicle. The time associated with the mean residual distance error is *t*_ε_ such that

(8) $$t_{\varepsilon }=\frac{d_{\mathrm{GT}}-d_{\mathrm{RES}}-d_{\mathrm{AX}}}{S}$$

where *S* is the average traversal speed for the GPS block containing the PIE.

Table 4 lists the values for *t*_ε_ calculated for each smartphone. The negative time bias suggests that the smartphone incurred a time latency in tagging accelerometer values with GPS coordinates. 

This is intuitive because the operating system (OS) of a smartphone must take a finite amount of time to calculate the GPS coordinates, fetch those values from an output register after receiving an interrupt, and then store those values to memory along with the accelerometer, speed, and timer samples. This latency is likely to vary among smartphones, depending on the number of process threads running, their processor speeds, and their data bus speeds. From Table 3, the average value of the GPS tagging latency across all experiments was 0.27 s with a standard deviation of 0.19 s. There was no significant correlation of GPS tagging latency among smartphone models. This suggests that faster processors or bus speeds did not necessarily affect the GPS tagging latency. This suggests that there are other smartphone operational characteristics that cause the GPS tagging latency among smartphones. Identification of those factors will be the focus of future work.

## 4. Discussion

The nature of the two distance distributions infers a statistical model that can be used to estimate the position of an anomaly. The normal distribution of the GPS distance error suggests that the centroid of the geospatial coordinates of the GPS blocks containing the PIE would be a good starting point for the estimator. Furthermore, computing a centroid enables the detection and removal of outliers due to non-linear errors such as multipath reflections. The latitude and longitude coordinate pair (*θ*_i_, *Φ*_i_) of a centroid computed from the geospatial positions of *N* GPS blocks containing PIEs is

(9) $$\left(\theta ,\varphi \right)=\left(\frac{1}{N}\overset{N}{\underset{i=1}{\sum }}\theta_{i},\frac{1}{N}\overset{N}{\underset{i=1}{\sum }}\Phi_{i}\right)$$

Table 4 lists the geodesic distance from the centroid of each experiment to its respective ground truth as *C_GT_*. A normal distribution of the GPS distance error suggests that the confidence interval of the centroid position is ever-increasing with traversal volume *N*. Whereas this method is well suited for participatory sensing vehicles, it is less suitable for situations where only a single or relatively few traversals are available. This participatory sensing approach is robust to GPS errors because it leverages a large volume of traversal data to remove outliers and to continuously increase the precision of estimating a centroid location. For statistical significance and confidence in computing the centroid, the number of traversals used in practice should be at least 30.

From the error model of Equation (1), the estimated position of the anomaly is a distance offset *D*_X_ from the centroid, in the direction of travel and along the traversal path such that

(10) $$D_{X}=-D_{\mathrm{GT}}=-S\left(T_{\varepsilon }+T_{\mathrm{RES}}\right)-D_{\mathrm{AX}}$$

where *T*_ε_ is the mean time latency of GPS tagging. Both *T*_ε_ and *T*_RES_ are time delays; thus, they are negative values. Therefore, if *D*_AX_ is zero, the estimate will be a positive value, which is a distance ahead of the centroid position. If the mean GPS tagging latency is zero, the estimated position of the anomaly will still be a positive distance away from the centroid position.

The uniform distribution of the PIE position within a GPS block points to the midway position as the best estimate of that error component. This position is equivalent to the product of one-half the mean GPS update interval and the average speed *S* for the GPS block containing the PIE. Therefore, the position of the anomaly relative to the centroid position can be estimated as

(11) $$D_{X}=-S\left(T_{\varepsilon }+\frac{1}{2}\mu T_{\mathrm{GPS}}\right)-D_{\mathrm{AX}}$$

where *µT*_GPS_ is the mean update period of the GPS receiver. All the variables of this equation are deterministic. Table 4 lists the estimated distance *D*_X_ from the computed centroid position by using the values of *T*_ε_ estimated for each smartphone. The distance *C_GT_* from the centroid to the ground truth was measured. Hence, the error *E*_X_ of the distance estimates was determined, and the results are listed in Table 4.

The mean error of the distance estimate across all devices was 2 cm, and the standard deviation was 78 cm. For all experiments of this study, the mean of the standard deviations of the traversal speeds was 0.4 m∙s^−1^. The standard deviation of the distance estimate is proportional to the choice of speed interval from which the system selects traversal data. The recommended practice is to determine the mean traversal speed of a detected anomaly and then select traversal data that is within a few m∙s^−1^ of the mean to satisfy the confidence interval desired for the application. The confidence interval *CI* of the distance estimate is

(12) $$CI=D_{X}\pm Z_{\alpha }\frac{\sigma_{X}}{\sqrt{N}}$$

where *Z*_α_ = 1.96 for a 95% confidence interval, *σ*_X_ is the standard deviation of the distance estimate, and *N* is the number of traversals used. From Equation (11), *σ*_X_ depends on the standard deviations of the traversal speed, the time latency of GPS tagging, and the GPS update interval. Hence, the confidence interval of the estimate will increase in proportion to the standard deviation of the speed interval used for data selection and decrease by the square root of the number of traversals used.

A limitation of this approach is that, without further tests, the mean GPS tagging latency of a device is unknown. At relatively low speeds, the average latency may be sufficiently small to ignore because the ground truth will be within sight distance. However, this may not be the case at highway speeds. Determining the latency will require experiments like those described in this paper. However, such experiments are very time-consuming, and they must be conducted for each smartphone used in practice. Another alternative is to guess a time latency and then correct for it after discovering the actual position of ground truths with known coordinates. Such ground truths can be manhole covers, utility covers, speed cushions, drainage provisions, crowned intersecting streets, and sudden grade changes. It is also possible to write software to measure the average latency in tagging accelerometer samples with GPS positions. Those developments will be the focus of future work.

## 5. Conclusions

Roadway anomalies such as potholes, frost heaves, swelling, and cracking can worsen rapidly with dynamic vehicle loading and weather cycles. Therefore, transportation agencies need to frequently scan the network for anomalies and repair them before they cause safety issues and vehicle damage. Current methods of scanning for anomalies are too expensive to scale for more frequent and network-wide coverage. Consequently, many studies emerged to develop methods of using regular vehicles to measure ride quality data. However, regular vehicles must have the appropriate devices on board to enable a practical solution. Future deployments of connected vehicles will have all the required communications, computing, and sensing capabilities including a low-cost GPS receiver, an accelerometer, a speed sensor, and a high-resolution timer. Meanwhile, researchers and commercial software app providers used smartphones to evaluate different methods that use their embedded low-cost GPS receivers, accelerometers, and timers.

One finding of this research is that low-cost GPS receivers produce a large error spread in anomaly localization when collecting data with smartphones on board vehicles. The authors conducted 12 sets of experiments with many traversals of an isolated rough spot in four different environments. The longitudinal GPS error spread ranged from 21 m to 32 m, and the lateral error spread ranged from 9 m to 15 m. The experiments showed that the geodesic distance error increased linearly with traversal speed. The error exceeded 16 m at arterial speeds of 18.8 m∙s^−1^ (42 mph). A regression of the trend suggested that the error can exceed 27 m at highway speeds of 31.3 m∙s^−1^ (70 mph). Such a large error will be beyond the human sight distance.

This work contributes a method to estimate the position of anomalies with greater accuracy when using low-cost GPS receivers on board regular vehicles. The method leverages the large volume of participatory sensing data to calculate an outlier-free centroid of the GPS position tags of a peak inertial event (PIE) produced in the accelerometer signal while traversing an anomaly. The estimated position is a simple function of the average traversal speed, the GPS update interval, and the system latency in tagging accelerometer samples with GPS coordinates. The experiments determined that, with a good estimate of the system latency, the method can provide sub-meter accuracy. Future work will explore methods of automatically estimating the system latency. This will lead to the development of a server-side application that can provide ever-increasing accuracy of estimating the position of anomalies using connected vehicles.

## Figures and Tables

**Table 1:** Format of sensor data stream.

Time	Gz	Speed	Latitude	Longitude
44.142	−1.057	9.586	45.263	−93.711
46.768	−1.216 ^1^	9.586	45.263	−93.711
50.260	−1.087	9.586	45.263	−93.711
62.927	−0.854	9.586	45.263	−93.711
73.909	−0.912	9.586	45.263	−93.711
86.754	−0.942	9.586	45.263	−93.711
95.669	−1.001	9.586	45.263	−93.711
110.365	−1.022	9.586	45.263	−93.711
118.253	−1.096	9.586	45.263	−93.711
128.695	−1.013	9.586	45.263	−93.711

^1^ Peak inertial event (PIE).

**Table 2:** Equipment and environment of the experiments. SUV—sport utility vehicle.

Road	Environment and Rough Spot	Sensor Label	Phone Model	Speed (m∙s^−1^)	Vehicle Type and Sensor Mount
MnRoad	Concrete panel road, open environment, few trees, slightly overcast, joint bump	i4S1	iPhone^®^ 4S	18.8	2011 Chevy Traverse minivan; behind the rear axle, taped flat onto the trunk floorboard, near the vehicle center line
		i4S2	iPhone^®^ 4S	18.8	
		i5	iPhone^®^ 5	18.8	
Unpaved Road	Gravel road, open environment, no trees, snow covered adjacent land, dawn, rail tracks	i8	iPhone^®^ 8	10.9	2015 Volkswagen Jetta sedan; midway between the vehicle axles, taped flat onto the floorboard on the passenger side
		iX	iPhone^®^ 10	11.2	
		GP	Google Pixel	11.3	
Paved Road	Asphalt road, open environment, few trees, dusk, rail tracks	i8	Identical smartphones and set-up as for the Unpaved Road	11.8	Identical set-up as for the Unpaved Road
		iX		11.6	
		GP		11.5	
Park Road	Asphalt road, tree lined park, clear midday, speed bump	i4_a	iPhone^®^ 4	2.5	2001 Ford Explorer SUV; behind the front axle, taped flat in a tray between the driver and passenger seats.
		i4_b	iPhone^®^ 4	5.0	
		i4_c	iPhone^®^ 4	7.5	

**Table 3:** Parameters measured from the experiments.

Road	Sensor Label	*N*	*µ*_s_ (Hz)	*S* (m∙s^−1^)	*D*_GT_ (m)	*D*_AX_ (m)	*D*_RES_ (m)	*T*_RES_ (s)	*t*_ε_ (s)	*D*_GT_ *p*-value	*D*_RES_ *p*-value
**MnROAD**	i4S1	17	91.2	18.8	−16.5	−3.6	8.84	0.47	−0.22	0.94	0.96
	i4S2	17	91.3	18.8	−16.7	−3.6	10.07	0.54	−0.16	0.55	0.33
	i5	17	137.4	18.8	−15.7	−3.6	10.03	0.53	−0.11	0.80	0.61
**Unpaved** **Road**	i8	30	132.8	10.9	−10.5	1.3	5.63	0.51	−0.57	0.48	0.61
	iX	34	133.8	11.2	−7.0	1.3	5.93	0.55	−0.21	0.11	0.35
	GP	31	385.4	11.3	−6.2	1.3	6.30	0.59	−0.11	0.60	0.42
**Paved** **Road**	i8	35	132.8	11.8	−11.0	1.3	6.28	0.53	−0.52	0.58	0.77
	iX	34	133.8	11.6	−7.7	1.3	6.50	0.55	−0.22	0.66	0.90
	GP	35	386.5	11.5	−10.4	1.3	6.19	0.53	−0.48	0.54	0.72
**Park** **Road**	i4_a	29	93.2	2.5	−2.8	−0.9	1.39	0.56	−0.17	0.82	0.20
	i4_b	28	93.2	5.0	−6.5	−0.9	3.34	0.66	−0.45	0.66	0.20
	i4_c	30	93.2	7.3	−2.6	−0.9	3.92	0.54	0.32	0.25	0.74
**Average**								0.55	−0.24	0.58	0.57
**SD**								0.05	0.24	0.23	0.26

**Table 4:** Prediction of anomaly position from centroid.

Road	Sensor Label	*S* (m∙s^−1^)	*C*_GT_ (m)	*D*_AX_ (m)	*t*_ε_ (s)	*D*_X_ (m)	*E*_X_ (m)
**MnROAD** **Cell 40**	i4S1	18.8	−16.08	−3.6	−0.22	−17.10	−1.02
	i4S2	18.8	−16.53	−3.6	−0.16	−15.99	0.54
	i5	18.8	−15.52	−3.6	−0.11	−15.11	0.42
**Unpaved** **Road**	i8	10.9	−10.37	1.3	−0.57	−10.36	0.01
	iX	11.2	−6.35	1.3	−0.21	−6.65	−0.30
	GP	11.3	−5.90	1.3	−0.11	−5.52	0.38
**Paved** **Road**	i8	11.8	−10.96	1.3	−0.52	−10.64	0.32
	iX	11.6	−7.56	1.3	−0.22	−7.01	0.56
	GP	11.5	−10.10	1.3	−0.48	−9.93	0.18
**Park** **Road**	i4_a	2.5	−3.06	−0.9	−0.17	−2.63	0.43
	i4_b	5.0	−6.41	−0.9	−0.45	−5.71	0.70
	i4_c	7.3	−2.59	−0.9	0.00	−4.57	−1.98
**Average**					−0.27		0.02
**SD**					0.19		0.78
